# Docetaxel-induced photolichenoid eruption

**DOI:** 10.4103/0253-7613.56065

**Published:** 2009-08

**Authors:** Biju Vasudevan, M.P.S. Sawhney, Nitu Sharma

**Affiliations:** Department of Dermatology, Base Hospital, Delhi Cantt, New Delhi, India; 1 Department of Pathology, Base Hospital, Delhi Cantt, New Delhi, India

**Keywords:** Adverse effects, docetaxel, lichenoid eruption

## Abstract

A 58-year-old man presented with complaints of blackish discoloration of forearms and face of five months duration. The lesions occurred episodically after taking anti-cancer medications, each episode lasting for two weeks. Histopathology confirmed a lichenoid eruption. Photolichenoid eruption to docetaxel is a dermatological adverse effect not reported in literature earlier.

## Introduction

Docetaxel is a clinically well established antineoplastic medication used mainly for the treatment of breast, ovarian, prostate and non-small cell lung cancer.[1] Docetaxel acts by stabilizing microtubules, enhancing the rate and extent of tubulin polymerization and inhibiting depolymerization. The main toxic effects of the drug are neutropenia, fluid retention, asthenia, neurotoxicity, hypersensitivity and cutaneous reactions.[2] We herein describe a case of photolichenoid eruption as a side effect of docetaxel, which has not been reported in literature earlier.

## Case Report

A 58-year-old male on treatment for prostate cancer since two years, presented to the skin OPD with complaints of dark colored raised lesions on hands and face of three days duration. He initially noticed lesions on the face which spread to involve the neck and both forearms and hands. The lesions were associated with moderate to severe itching which increased on sun exposure. There was history of such episodic appearance of lesions for the last four to five months. He was taking docetaxel injections for prostate cancer once in three weeks since five months. The lesions had initially appeared two to three days after the first injection, according to his case history. Subsequently they would fade in two weeks but again flare up after the next injection. The patient was not administered any other drug simultaneously for the same disease or for any other associated or non associated disease condition.

**Figure 1:**
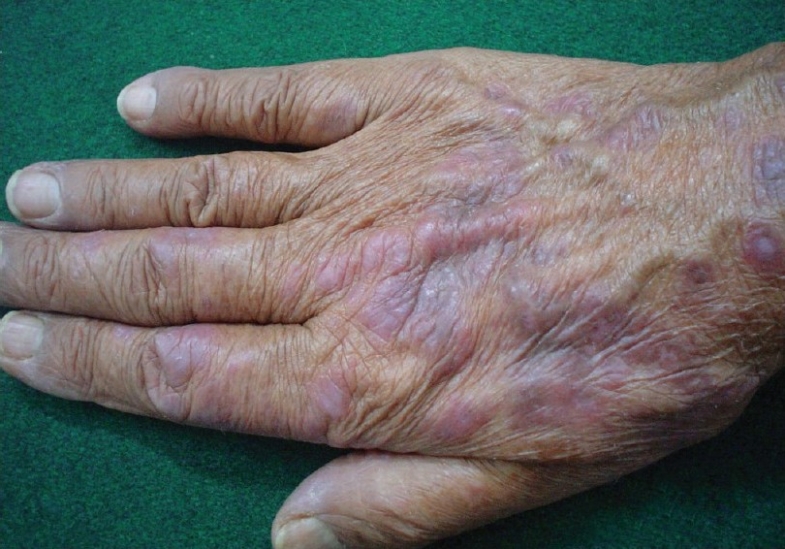
Photolichenoid lesions on hands

**Figure 2:**
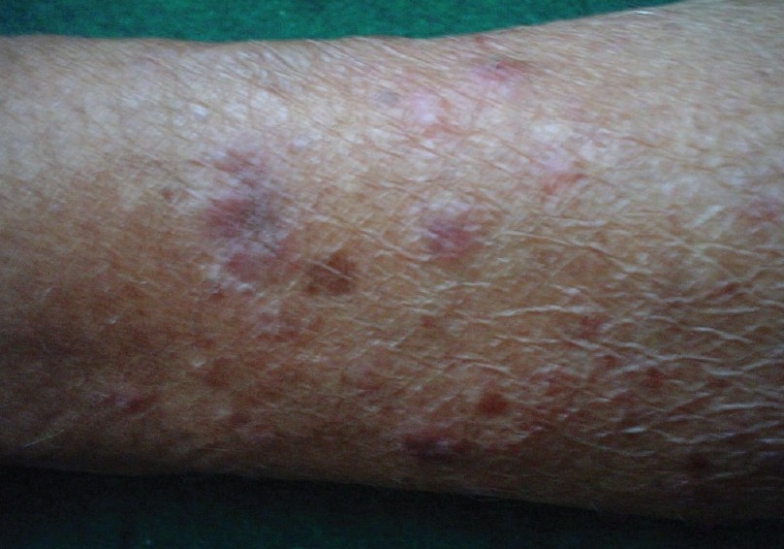
Lesions on photo-exposed parts of forearm

**Figure 3:**
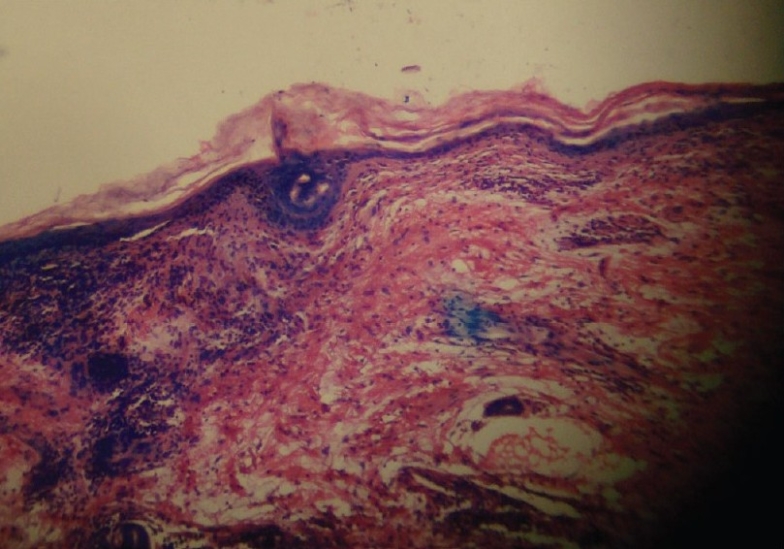
Histopathology showing lichenoid reaction

On examination, violaceous, well defined, non scaly, non tender plaques were present on the dorsum of hands, extensor surface of forearms, face and front of neck [Figures 1 and 2]. Only the photo exposed parts of the face were involved. Systemic examination was within normal limits. A clinical diagnosis of drug induced photolichenoid eruption with differential diagnosis of discoid lupus erythematosus was kept in mind. On investigating, the patient had microcytic hypochromic anemia with hemoglobin of 9.8gm%. The differential white blood cell (WBC) count showed increased eosinophils with an absolute eosinophilic count of 850/mm^3^. All other hematological and biochemical parameters were within normal limits. ANA and dsDNA were repeatedly negative. Skin biopsy revealed a band like lymphocytic infiltrate along the dermo epidermal junction along with abundant melanophages and a mixed eosinophilic-lymphocyte infiltrate in dermis [Figure 3]. A diagnosis of photolichenoid eruption to docetaxel was thus confirmed. The medication was stopped and replaced by estramustine phosphate. Sunscreen, topical corticosteroid cream and tab Avil were prescribed. The lesions regressed in one week. The patient has been observed for six months following change of therapy. No fresh lesions have been observed.

## Discussion

Docetaxel is of the chemotherapy drug class taxane and is a semi-synthetic analogue of Paclitaxel, an extract from the rare Pacific yew tree Taxus brevifolia. It has the empiric formula C_43_H_53_NO_14_.3H_2_O with a molecular weight of 861.9. Docetaxel differs from paclitaxel at two positions in its chemical structure. It has a hydroxyl functional group on carbon 10, whereas paclitaxel has an acetate ester and a tertiary-butyl substitution exists on the phenyl propionate side chain. The carbon 10 functional group change causes docetaxel to be more lipid soluble than paclitaxel. Intravenous administration of docetaxel results in 100% bioavailability and absorption is immediate. Administered as a one-hour infusion every three weeks, generally over a 20 cycle course, it has a half life of 11-18 hrs and is metabolized in the liver by cytochrome P450-3A.[3,4] About 80% of elimination is through the feces while five per cent is excreted in urine; 95% of the drug is bound to plasma proteins.

As docetaxel is a cell cycle specific agent it is cytotoxic to all dividing cells in the body[5] and hence exhibits cytotoxic activity on breast, colorectal, lung, ovarian, gastric, renal and prostate cancer cells. Docetaxel has also been found to have greater cellular uptake and is retained longer intracellularly than paclitaxel allowing docetaxel treatment to be effective with a smaller dose, leading to fewer and less severe adverse effects. Docetaxel is contraindicated for use with patients with; baseline neutrophil count less than 1.5 × 109 cells/L, history of severe hypersensitivity reactions to docetaxel or polysorbate, severe liver impairment and pregnant or breast-feeding women. Erythromycin, ketoconazole and cyclosporine are CYP3A4 inhibitors and therefore inhibit the metabolic pathway of docetaxel. When used with anticonvulsants which induce CYP3A4 an increased dose of docetaxel may be required. Elderly people are especially sensitive to the effects of docetaxel. This may increase the chance of side effects during treatment.

Major systemic side effects of docetaxel include bone marrow suppression, hypersensitivity reactions, fluid retention, neurotoxicity, myositis and cardiovascular side effects. Hematological adverse effects include neutropenia, anemia, febrile neutropenia and thrombocytopenia. Hypersensitivity reactions can range from minor rash to severe anaphylaxis. Fluid retention can take the form of generalized edema, pleural effusion and ascites, all of which are reversible on stopping treatment. Other rare effects include vomiting, diarrhea, asthenia and arthralgias. Premedication with Oral corticosteroids is advised starting from a day before infusion and lasting for four to five days to prevent these adverse effects.

Skin reactions mentioned in literature include maculopapular drug rash, supravenous discoloration, mucosal ulcers, alopecia, nail hyper pigmentation and destruction.[6] Docetaxel may also produce a potentially life-threatening, toxic epidermal necrolysis-like adverse reaction. In our case the patient had photolichenoid eruption following the cyclic administration of docetaxel. This was confirmed clinically and by the classic histopathology. Such a dermatological adverse effect of the drug has not been mentioned in literature earlier. As docetaxel is now a widely used antineoplastic drug, clinicians should take into consideration the above-described side effect while prescribing it.
